# Psychological assessment in patients suffering from chronic pain treated with Spinal Cord Stimulation: A prospective observational study protocol

**DOI:** 10.1371/journal.pone.0352398

**Published:** 2026-07-14

**Authors:** Chiara Marzorati, Massimo Pezzolato, Florence Didier, Federico Borgogni, Cristiana I. Fodor, Sara Meneghin, Vittorio A. Guardamagna, Gabriella Pravettoni

**Affiliations:** 1 Applied Research Division for Cognitive and Psychological Science, European Institute of Oncology, IRCCS, Milan, Italy; 2 Department of Oncology and Hemato-Oncology, University of Milan, Milan, Italy; 3 Division of Radiation Oncology, European Institute of Oncology, IRCCS, Milan, Italy; 4 Division of Palliative Care and Pain Therapy, European Institute of Oncology, IRCCS, Milan, Italy; University of Catania, ITALY

## Abstract

**Introduction:**

Spinal cord stimulation (SCS) has been used to manage a variety of chronic pain conditions, including cancer population. People suffering from this medical condition often report strong limitations in their daily and work activities, and a deterioration of health-related quality of life. This study aims at investigating the relationship between psychological factors and intervention outcomes (pain reduction, removal of SCS). Further, the acceptability and satisfaction levels regarding the SCS intervention will be assessed.

**Methods and analysis:**

Cancer survivors 1) suffering from chronic pain (VAS > 4) from at least 6 months, 2) aged over 18 years, and 3) refractory to conventional pain therapies will be assessed at the pre-surgical visit (T0), and after 1, 2, 3, 5, and 8 months from T0. During each assessment, patients will undergo a clinical consultation on their ability to cope with chronic pain and fill out validated questionnaires on patients’ perception of pain (BPI, PCS), state and trait anxiety (STAI Y1-Y2), depression (BDI-II), and pain-specific resilience (PRS). Patients Global Impression of Change (PGIC) after SCS implantation will be also assessed. Data collection is ongoing. The correlation between continuous variables at considered time points will be evaluated with Pearson’s correlation coefficient. The difference in mean patients’ pain, anxiety, depression, catastrophizing tendency, and resilience according to the SCS removal request will be tested with Student’s t-test. Finally, the overall correlation between a psychological trait of interest and an intervention outcome over the entire course of observation will be estimated using a bivariate linear mixed effect model for longitudinal data.

**Ethics and dissemination:**

The study protocol was reviewed and approved by the Ethics Committee of the European Institute of Oncology (Comitato Etico Territoriale Lombardia 2; R1865/23 – IEO 2028). The results of the study will be disseminated through a peer-reviewed journal.

**Registration details:**

The study is registered on ClinicalTrials.gov (Identifier: NCT06761300).

## Introduction

Chronic pain (CP), commonly defined as “an unpleasant sensory and emotional experience associated with actual or potential tissue damage, or described in terms of such damage”, affect more than 30% people around the world [1,2]. Its prevalence and impact at societal and individual levels led the World Health Organization (WHO) to reclassify this health problem from symptom to disease [3]. Neuropathic CP comprises a large and diverse array of intermittent or persistent physical symptoms combining allodynia, paresthesia, and hyperalgesia symptoms [4,5].

People suffering from this medical condition often report strong limitations in their daily and work activities, insomnia, and a consequent deterioration of health-related quality of life [6–8]. Also, cognitive and emotional factors, individual perceptions, beliefs, and expectations greatly influence the experience of CP and deeply affect patients’ social and environmental contexts [9,10]. A meta-analysis on the psychological functioning of people living with CP showed that feelings directly related to the physical condition (i.e., pain concern and somatization) and mood disorders significantly affect patients’ well-being: anxiety and depression symptoms, as well as self-efficacy problems, were systematically more present in individuals with CP than in healthy subjects [11].

Therefore, the management of CP is based on a multidisciplinary approach that combines traditional and holistic medicine to compensate for the refractoriness of conventional treatments and reduce the related toxicity and the impact on quality of life [5,12–14]. CP clinical path often includes opioid administration, psychological counseling, acupuncture, and physical therapies, but even the combination of all these therapies cannot always relieve neuropathic pain [15]. In this scenario, the surgical implantation of spinal cord stimulation (SCS) is the last promising adjuvant therapy in controlling CP [16]. Indeed, numerous clinical trials showed the effectiveness of this technique in consistently reducing pain symptoms, especially in those conditions in which other treatments did not give any promising results [17–19]. The fact that, after SCS implantation, 30–80% of patients reported ≥ 50% reduction in perceived pain levels compared to pre-surgical condition, highlights the potentially positive effect of this procedure. The high variability in the outcomes of the SCS implantation could partially be explained by the great influence of individual and relational characteristics of patients (e.g., gender, psychosocial aspects, coping strategies) in CP experience [18]. Indeed, in a systematic review examining the relationship between pre-surgical predictors and pain-related treatment outcomes in patients undergoing SCS procedure, most of the included studies (92%) reported that somatization, coping strategies, depression, and anxiety symptoms predict a reduced perception of treatment’s benefits [20]. Moreover, the presence of psychiatric comorbidities and long-lasting pain symptoms are relevant risk factors for poorer perceived benefits and may affect patients’ decision to remove the SCS [20,21].

The relevance of these aspects is even more evident when we consider that high levels of psychological distress, pain-related anxiety and fear, and a great impact of CP on daily and social activities are commonly reported by SCS candidates [22].

The great impact of all these aspects along the process of SCS implantation highlighted the need to better investigate the relationship between psychological factors and the management of CP before and after this surgical procedure, to understand better how these variables may affect the acceptability of the SCS implantation.

Despite the results previously reported, several clinical issues related to device removal and patients’ satisfaction are still relevant.

To this end, the present study aims to evaluate patients’ perception of CP before and after the SCS surgical procedure and assess how the emotional status affects this medical iter. Moreover, the 6 months follow-up planned for this study would allow a thorough detection of possible changes in patients’ clinical and psychological outcomes over time.

## Materials and methods

### Primary aim

The primary aim is to study the associations between psychological variables and intervention outcomes (pain reduction, satisfaction, removal of SCS). We hypothesize that people with higher scores in anxiety and depression symptoms would report lower satisfaction with the SCS procedure and higher levels of perceived pain after the implantation of the device. Moreover, patients with higher levels of resilience may show lower removal requests. Finally, a greater tendency in catastrophizing pain symptoms may result in higher removal rates and lower levels of satisfaction with SCS.

### Secondary aims

Additionally, this study will explore the acceptability and satisfaction levels regarding the SCS treatment and how this surgical procedure may affect patients’ perception of CP. Our hypothesis suggests that patients who report minimal improvement in CP levels may be more likely to request the removal of the device. Lastly, we will examine the perceived benefits of psychological counseling throughout the care pathway.

### Endpoints

The following endpoints are assessed with self-report measures: pain intensity and pain interference (BPI), changes in perceived chronic pain (PGIC), anxiety (STAI-Y), depression (BDI-II), pain catastrophizing (PCS), and pain resilience (PRS).

Other endpoints are removal request (categorical: yes/no), and perceived benefit of psychological counseling (qualitative: open answer).

### Study design

This is a prospective observational study to investigate the impact of the SCS procedure on CP perception and management. Patients’ psychological status at different time points and its correlation with pain outcomes and satisfaction with the SCS intervention is also evaluated.

The study is registered on ClinicalTrials.gov (Identifier: NCT06761300).

### Sample size

Considering that, on average, around 20–25 patients annually are admitted to the Palliative Care and Pain Therapy Division of the IEO for surgical implantation of an SCS to alleviate their pain symptoms, we anticipate enrolling a total of 50 patients within a 2-year study period. Assuming that the correlation between a psychological trait of interest (e.g., anxiety, measured by the STAI-Y) and an intervention outcome (e.g., pain intensity, measured by the BPI) at a specific time point is at least 0.4, the expected sample size will guarantee, with 80% of power, to reject the null hypothesis of no correlation with a 5% type I error. Given the exploratory nature of this study, no adjustment for multiple comparisons will be performed.

### Patients population

Patients are being involved based on the following inclusion and exclusion criteria:

#### Inclusion and exclusion criteria.

The inclusion criteria for participants in the study are as follows: I) individuals experiencing CP, with a Visual Analog Scale (VAS) score greater than 4, persisting for a minimum of six months, II) who are eligible for SCS implantation, III) aged 18 years or older, IV) demonstrating willingness and capability to adhere to scheduled appointments and other trial-related procedures, V) speaking fluent Italian, and VI) who agree to sign a written informed consent before participation in the study. Patients with psychiatric disorders or conditions that might impair the ability to give informed consent, as well as with comorbidity that may impact compliance to study procedures will be excluded from the enrollment.

### Patient and public involvement

None.

### Patients’ enrollment

Patients suffering from CP in charge of the Palliative Care and Pain Therapy Division at the European Institute of Oncology IRCCS (Milan, Italy) are proposed to undergo SCS surgical implantation to mitigate pain symptoms.

The SCS surgical implantation is a two-step procedure; after the hospital admission, a temporary SCS device will be first implanted. In this way patients can experiment with the device’s impact on their CP conditions, familiarize themselves with the external device and adjust to its presence, and eventually grow their motivation toward the implantation of the permanent device. Only after one month, if the patients are convinced of the intervention’s utility, the temporary device will be changed to a permanent one.

After the medical consultation, patients accepting the surgical procedure and meeting the inclusion criteria are invited to participate in the study.

Patients signing the informed consent are considered enrolled and the first clinical assessment with the psychologist is scheduled.

### Data management plans

Socio-demographic and clinical data are collected at baseline.

The following validated questionnaires are administered (see Appendix):

#### Brief pain inventory (BPI).

The Brief Pain Inventory (BPI) is a self-report tool composed of 9 items. Originally developed to evaluate pain in oncologic patients, it is now commonly used also with patients suffering from different pain typologies [23]. The 9 items constituting BPI allow to measure 2 pain dimensions: 1) Pain Intensity which will be calculated from the sum of 4 items ranging from 0 (no pain) to 10 (pain as bad as you can imagine) and with a final score varying between 0 and 40; and 2) Pain Interference which will be calculated from the sum of 7 items ranging from 0 (does not interfere) to 10 (completely interferes) and with a final score varying between 0 and 70.

BPI’s psychometric properties proved to be good. Test-retest reliability is good for both domains (r = 0.8), and internal consistency results are high (0.81 < α < 0.89 for the severity scale, and 0.88 < α < 0.95 for the interference scale). The 2-factors structure presents good construct validity, whilst, pertaining to criterion validity, correlation resulted moderate both with the SF-36 Bodily Pain (0.47 < *r* < 0.65), and with the Roland Morris Disability Questionnaire (*r* = 0.57). Responsiveness indices (effect size, standardized response mean, and responsiveness index) were adequate [24].

#### Pain Catastrophizing Scale (PCS).

The Pain Catastrophizing Scale is a 13-item self-administered tool assessing pain catastrophizing. The items are answered with a 5-point Likert-type scale and the scores compose 3 sub-scales: rumination (score ranging from 0 to 20), magnification (score ranging from 0 to 8), and helplessness (score ranging from 0 to 24). A total score is calculated by summing the 3 sub-scales (ranging from 0 to 52).

Reliability analysis highlighted moderately acceptable reliability with *α* indices of 0.93, 0.91, 0.75, and 0.87, respectively for the total score and the 3 sub-scales [25]. Test-retest reliability was found to be acceptable over 6 weeks (*r* = 0.75) and over 10 weeks (*r* = 0.70) [26]. A significant positive correlation with the Inventory of Negative Thoughts in Response to Pain testifies to PCS’ convergent validity [25].

#### Patients Global Impression of Change (PGIC).

The Patients Global Impression of Change is a brief self-report tool commonly used in routine clinical practice to assess the perceived change in pain experience after starting the treatment. The first 7-point item evaluates patients’ impression of change ranging from “very much worse” to “very much improved”. The second item evaluates the perceived change on a numerical scale ranging from 0 (“much better”) to 10 (“much worse”).

This instrument’s validity was verified in other clinical populations [27,28]. Recently, Perrot et al [27] reported significant correlations between PGIC and other well-established measures of pain intensity, and pain interference in daily life. and disease management efficacy, with a sample of neuropathic pain patients.

#### Beck Depression Inventory II (BDI-II).

The Beck Depression Inventory II is one of the most popular self-report measures for depression [29,30]. It is composed of 21 items measuring depressive symptoms and each item is rated on a 4-point Likert-type scale ranging from 0 to 3, based on the severity in the last 2 weeks. The total score ranges from 0 to 63.

BDI-II’s internal consistency is reported to be good, with Cronbach’s *α* coefficients ranging from 0.83 to 0.96. Good to excellent coefficients (0.73 < r < 0.96) are reported for what concerns test-retest reliability. The correlation between BDI-II and other depression and anxiety measures is high, suggesting a good convergent validity. Finally, BDI-II sensitivity and specificity are reported to be high, testifying to the inventory’s ability to assess depression [30].

#### State-Trait Anxiety Inventory (STAI-Y).

The State-Trait Anxiety Inventory is a self-report measure commonly used to assess anxiety. It is composed of 2 sub-scales of 20 items each, which allow to measure acute (state) and chronic (trait) anxiety. The items are rated with a 4-point Likert-type scale, and the total scores of each scale range from 20 to 80. STAI-Y internal consistency and overall item characteristics were reported to be good [31,32].

#### Pain Resilience Scale (PRS).

The Pain Resilience Scale is a 14-item self-report measure of pain-specific resilience. Each item is rated on a 5-point Likert-type scale, and the total score ranges from 0 to 56.

Test-retest reliability, construct validity, and internal consistency reliability were reported to be good [33]. The data available in the literature suggest that PRS can be of use in predicting people’s responses both to acute and CP [34].

### Patients’ assessment

Patients are assessed based on the following time points:

1) **Pre-surgical Assessment (T0)**

A psychologist evaluates the patient’s psychosocial status investigating the history of pain, how it affected psychological status, and social support. Moreover, patients’ resources in managing CP sequelae are assessed. At the end of the clinical consultation, the practitioner gives patients an information booklet about the SCS surgical intervention, the description of the two-step procedure, and any side effects.

After the consultation, patients receive a link with a set of questionnaires investigating patients’ perceptions of pain, state and trait anxiety, depression, and resilience. Moreover, the following questionnaires are administered: Brief Pain Inventory (BPI), Pain Catastrophizing Scale (PCS), Beck Depression Inventory II (BDI-II), State-Trait Anxiety Inventory (STAI-Y), and Pain Resilience Scale (PRS).

2) **Temporary SCS implantation (T1)**

After 2–4 weeks from T0, at the pre-surgical admission for the temporary SCS surgical intervention, a psychologist evaluates patients’ coping strategies and beliefs on the temporary SCS surgical procedure. The practitioner also assesses patients’ comprehension of the SCS implantation iter, and their expectations and he/she returns questionnaires’ results. The psycho-emotional status related to the imminent intervention is also explored and, if a significant amount of anxiety or worries is detected, further psychological support is provided.

3) **Permanent SCS implantation (T2)**

After one month from T1, a psychologist meets the patient during the pre-admission to implant the permanent SCS device. The practitioner assesses the patient’s pain perception and management and administers the following questionnaires: Brief Pain Inventory (BPI), Pain Catastrophizing Scale (PCS), and Patients Global Impression of Change (PGIC). Moreover, the perceived benefit of the provided psychological counseling is inquired with the following item: “Do you think that the psychological sessions have helped support you along the care pathway? If yes, in which aspects of your experience was it helpful?”.

4) **Follow-up time points (T3, T4, and T5)**

At 1, 3, and 6 months from T2, patients receive a link with the following questionnaires: Brief Pain Inventory (BPI), Pain Catastrophizing Scale (PCS), Beck Depression Inventory II (BDI-II), State-Trait Anxiety Inventory (STAI-Y1), and the Patients Global Impression of Change (PGIC). Additionally, the Pain Resilience Scale (PRS) is administered at T3 and T5. Moreover, the perceived benefit of the provided psychological counseling is inquired with the following item: “Do you think that the psychological sessions have helped support you along the care pathway? If yes, in which aspects of your experience was it helpful?”.

### Statistical analyses

Descriptive statistics of the sample and the questionnaires’ scores will be computed. Pearson’s correlation coefficient will be used to evaluate the correlation between continuous variables at considered time points. Student’s t-test will be used to test the difference in mean patients’ pain, anxiety, depression, catastrophizing tendency, and resilience according to the SCS removal request. A bivariate linear mixed effect model for longitudinal data will be fitted to estimate the overall correlation between a psychological trait of interest and an intervention outcome over the entire course of observation [35].

All analyses will be carried out with the SAS software (SAS Institute, Cary, NC).

### Ethics and dissemination

The study protocol was reviewed and approved by the Ethics Committee of the European Institute of Oncology (Comitato Etico Territoriale Lombardia 2; R1865/23 – IEO 2028). All the procedures are carried out in compliance with the Declaration of Helsinki. Participants provide written informed consent prior to enrollment.

The results of the study will be disseminated through a peer-reviewed journal.

### Status and timeline of the study

Patients recruitment started on the 9^th^ of April 2024, and the data collection is still ongoing. As outlined in the Sample size section, we expect enrolling a total of 50 patients within a 2-year study period.

## Expected results

Patients’ psychological well-being, the impact of CP in daily activities and the decision-making process to undergo to a surgical procedure will be analyzed as soon as the data collection will be completed.

## Discussion

The present protocol aims to explore the relationship between psychological factors and the outcomes of SCS intervention, including pain reduction, satisfaction levels, and the removal of SCS.

Previous studies have highlighted the significant influence of these factors along the process of SCS implantation, thus stressing the importance of thoroughly exploring the connection between emotional and individual characteristics and the management of CP, both before and after this surgical procedure.

Collecting information regarding not only medical variables but also patients’ attitudes and well-being may help healthcare providers and the overall care system to better understand factors contributing to improved treatment outcomes and lower device removal [36–38]. Indeed, higher levels of emotional distress (e.g., anxiety, depression) may affect pain and contribute to the decision to request SCS removal due to dissatisfaction, intolerance of the procedure, or unmet expectations on pain reduction. Of note, previous studies reported that patients who required SCS removal were affected by high rates of major depression and anxiety [36,39]; further, feelings of helplessness in response to pain were particularly claimed by patients who did not attain successful pain relief through SCS, leading to the adoption of negative coping strategies [40,41]. Additionally, patients with a higher tendency of catastrophizing pain symptoms were more inclined to report greater pain intensity and lower quality of life and satisfaction with SCS [42], while lower levels of anxiety and catastrophizing symptoms have been associated with meaningful clinical improvement [43].

On the other hand, we expect higher resilience to pain may lead to greater pain reduction and lower probability of device removal; indeed, individuals with greater resilience may be better equipped to cope with and manage their pain, and they may show greater tolerance for possible feelings of discomfort associated with the device [44,45].

Finally, the psychological sessions will foster a better understanding of the SCS procedure, its risks and benefits, and the related psychological sequelae. Exploring the way patients are coping with CP and surgical implantation will enhance their awareness about the treatment and care path. As highlighted by clinical practice and scientific literature [46], educational needs play a significant role in shaping the pain experience and consequently influence treatment response. Feeling confident and well-informed about the treatment can enhance self-efficacy and refine patients’ expectations regarding harms and benefits, potentially improving overall outcomes and fostering the shared decision-making process [47]. In this framework, findings coming from this study, when adequately integrated in patient-physician communication, may also improve the quality and completeness of information provided to patients and their caregivers, as well as their understanding.

## Conclusion

The analysis of the set of variables considered in this study will guarantee a better understanding of how psychological variables might influence patients’ experiences before and after SCS implantation; the result will be a broader overview of this clinical issue, contributing to the development of more personalized recommendations. Finally, recognizing the clinical and psychological characteristics of those patients that will plausibly encounter greater difficulties in adapting to the SCS implantation, will allow the clinicians and healthcare professionals to offer targeted interventions, further supporting those who need it the most.

### Strengths and limitations of this study

- The assessment is being conducted at 6 time points distributed over 8 months, before and after SCS implantation, thus offering a relatively extended time-frame to properly evaluate the outcomes of interest;
- Focusing on both clinical and psychological variables will provide a deeper understanding of factors involved in achieving improved treatment outcomes and lower device removal;
- Psychological assessment requires time and resources, which may not be readily available in all hospitals, thus limiting the applicability of the study results.
- If adverse events or removal requests will occur after more than 6 months from the implantation of the definitive device, the current study will not be able to account for them.
